# Butyric Acid and Leucine Induce α-Defensin Secretion from Small Intestinal Paneth Cells

**DOI:** 10.3390/nu11112817

**Published:** 2019-11-18

**Authors:** Akiko Takakuwa, Kiminori Nakamura, Mani Kikuchi, Rina Sugimoto, Shuya Ohira, Yuki Yokoi, Tokiyoshi Ayabe

**Affiliations:** 1 Department of Cell Biological Science, Graduate School of Life Science, Hokkaido University, Kita-21, Nishi-11, Kita-ku, Sapporo, Hokkaido 001-0021, Japan; takakuwa@tenshi.ac.jp (A.T.); kiminori@sci.hokudai.ac.jp (K.N.);; 2 Department of Nutrition, Faculty of Nursing and Nutrition, Tenshi College, 3-1-30 Higashi, Kita-13, Higashi-ku, Sapporo Hokkaido 065-0013, Japan; 3 Department of Cell Biological Science, Faculty of Advanced Life Science, Hokkaido University, Kita-21, Nishi-11, Kita-ku, Sapporo, Hokkaido 001-0021, Japan

**Keywords:** Paneth cell, α-defensin, short-chain fatty acid, amino acid, innate immunity, intestinal microbiota, butyric acid, leucine, Gpr41, Slc7a8

## Abstract

The intestine not only plays a role in fundamental processes in digestion and nutrient absorption, but it also has a role in eliminating ingested pathogenic bacteria and viruses. Paneth cells, which reside at the base of small intestinal crypts, secrete α-defensins and contribute to enteric innate immunity through potent microbicidal activities. However, the relationship between food factors and the innate immune functions of Paneth cells remains unknown. Here, we examined whether short-chain fatty acids and amino acids induce α-defensin secretion from Paneth cells in the isolated crypts of small intestine. Butyric acid and leucine elicit α-defensin secretion by Paneth cells, which kills *Salmonella typhimurium*. We further measured Paneth cell secretion in response to butyric acid and leucine using enteroids, a three-dimensional ex vivo culture system of small intestinal epithelial cells. Paneth cells expressed short-chain fatty acid receptors, *Gpr41*, *Gpr43*, and *Gpr109a* mRNAs for butyric acid, and amino acid transporter *Slc7a8* mRNA for leucine. Antagonists of Gpr41 and Slc7a8 inhibited granule secretion by Paneth cells, indicating that these receptor and transporter on Paneth cells induce granule secretion. Our findings suggest that Paneth cells may contribute to intestinal homeostasis by secreting α-defensins in response to certain nutrients or metabolites.

## 1. Introduction

Paneth cells, one of the small intestinal epithelial cell lineages, secrete the antimicrobial peptide α-defensin and contribute to enteric innate immunity through microbicidal activities [1,2,3]. It has been reported that mouse α-defensin, cryptdin, elicits specific bactericidal activities, i.e., potent killing of pathogenic bacteria, with a lesser response against commensals [4]. Additionally, the small intestinal microbiota changes in response to a difference in α-defensin levels [5]. Secreted α-defensin influences the colonic microbiota based on a previous report, which showed that α-defensin was recovered from the colonic lumen and feces [6,7]. The human intestine harbors over 1 × 10^14^ diverse bacteria [8]. The intestinal microbiota composition and metabolites affect host immunity; therefore, a normal intestinal microbiota is important for intestinal homeostasis.

One layer of small intestinal epithelial cells constituting the crypts and villi is constantly exposed to stimulation from the intestinal microbiota and separates the intestinal tissue that is “inside” from the intestinal tract cavity, which is “outside” of the layer of small intestinal epithelial cells. It has been reported that abnormalities in α-defensin secretion by Paneth cells are involved in the onset and pathology of certain diseases, such as inflammatory bowel diseases, obesity, or graft-versus-host disease (GVHD) [9]. A decrease in the number of Paneth cells and morphological Paneth cell abnormalities lead to dysbiosis, the disruption of the intestinal microbiota, via a reduction or dysfunction in α-defensin secretion, resulting in the onset of certain diseases. Therefore, α-defensin secreted by Paneth cells maintains intestinal homeostasis, possibly by regulating the intestinal microbiota, which contributes to maintaining health and preventing disease [10].

Previously, Paneth cells were shown to secrete α-defensin in response to stimuli from cholinergic agents and bacteria, including live and dead bacteria, or their antigens, including lipopolysaccharide (LPS) [1]. We previously reported that α-defensin, secreted in the lumen of the mouse intestine, is highest in the ileum, where the number of intestinal bacteria increases dramatically, as well as in the duodenum and jejunum, where food digestion and absorption occur [11]. Additionally, a correlation between Paneth cell number and diet has been reported; the number of Paneth cells in the duodenum and jejunum decreases following excessive calorie intake (diet rich in carbohydrate and fat) [12]. Paneth cells are known to form a stem cell niche [13] and enhance stem cell function in response to calorie limitations [14,15]. Thus, Paneth cells may respond to the intestinal environment, such as dietary factors or the absorption process. However, it is not known whether α-defensin secretion by Paneth cells is induced by nutrients.

This study was conducted to clarify whether short-chain fatty acids (SCFAs) and amino acids, both of which are nutrient sources in the intestinal environment, induce α-defensin secretion by Paneth cells. Since the SCFAs and amino acids also have signal-transducing roles [16,17], we analyzed whether these nutrients trigger Paneth cell secretion. Here, we show that butyric acid and leucine induce α-defensin secretion from Paneth cells. We evaluated the effect of SCFA and amino acid on the induction of Paneth cell secretion using isolated crypts of the mouse small intestine. Additionally, we showed that Paneth cells express receptors and transporter that recognize butyric acid and leucine, suggesting that Paneth cells secrete α-defensin via these receptors and transporter. Using microinjection methods on enteroids [18], granule secretion in response to butyric acid and leucine was evaluated. Further, antagonists of Gpr41 and Slc7a8 inhibited butyric acid-induced and leucine-induced granule secretions from Paneth cells, respectively, suggesting involvement of these receptors and the transporter in Paneth cell secretion. This study suggests a potential new role for nutrients as intestinal environmental factors that maintain intestinal homeostasis by stimulating the secretion of α-defensin by Paneth cells.

## 2. Materials and Methods

### 2.1. Animals

Crlj: CD1 (ICR) mice were purchased from Charles River Japan (Kanagawa, Japan). All animal experiments were approved by the Committee of Animal Experimentation at Hokkaido University.

### 2.2. Reagents

Propionic acid (PA), butyric acid (BA), L-glycine (Gly), L-alanine (Ala), L-serine (Ser), L-threonine (Thr), L-cysteine (Cys), L-methionine (Met), L-valine (Val), L-leucine (Leu), L-isoleucine (Ile), L-phenylalanine (Phe), L-tryptophan (Trp), L-aspartic acid (Asp), L-glutamic acid (Glu), L-asparagine (Asn), L-glutamine (Glu), L-histidine (His), L-asparagine monohydrate (Arg), L-tyrosine (Tyr), and L-ascorbic acid were purchased from FUJIFILM Wako (Osaka, Japan). Acetic acid (AA), L-lysine (Lys), and sodium butyrate were purchased from Nacalai Tesque (Kyoto, Japan), and proline (Pro) was purchased from Sigma-Aldrich (St. Louis, MO, USA). All chemicals were of analytical grade.

### 2.3. Crypt Isolation

#### 2.3.1. Crypt Isolation for Sandwich Enzyme-Linked Immunosorbent Assay (ELISA) and Bactericidal Assay

Crypts were isolated from the small intestine, as previously described [1]. The small intestine lumen of adult mice was rinsed with ice-cold water, yielding the ileum segments. The segments were everted and shaken in cold Ca^2+^- and Mg^2+^-free Hank’s balanced salt solution (HBSS) containing 30 mM EDTA to detach the crypts. Villi and crypts detached for 7-min intervals were deposited by centrifugation at 700× *g* for 5 min at 4 °C and resuspended in phosphate-buffered saline (PBS). For experiments using ˃10,000 crypts, the numbers were estimated by hemocytometry.

#### 2.3.2. Crypt Isolation for Quantitative Polymerase Chain Reaction (qPCR) and Enteroid Culture

For crypt isolation, mouse small intestine was flushed with cold Ca^2+^- and Mg^2+^-free PBS and cut open lengthwise in ~10 cm long pieces. The villi were scraped off using a scalpel blade and washed with cold PBS. The tissue fragments were incubated in 30 mM EDTA with HBSS for 10 min at 25 °C. The solution was removed, and the tissue was shaken vigorously for ~300 times in fresh HBSS. Intact tissue was discarded, and dissociated crypts were pelleted by centrifugation at 440× *g* for 4 min at 4 °C.

### 2.4. Stimulation and Collection of Paneth Cell Secretions

The crypt fractions obtained in Section 2.3.1 were incubated at 37 °C for 30 min to stimulate secretion of a-defensin from Paneth cells by adjusting the final concentration to 100 µM SCFAs or 1 µM amino acids and PBS control. Supernatants were collected by centrifugation at 700× *g* for 5 min at 4 °C. Supernatants were adjusted to 30% acetic acid, and proteins were extracted using a 1000 Da dialysis membrane (Spectrum Laboratories, Rancho Dominguez, CA, USA) overnight at 4 °C. The solution after the dialysis was lyophilized and stored at −80 °C until use.

### 2.5. Sandwich ELISA

The materials obtained in Section 2.4 were dissolved in 200 µL of PBS, and cryptdin-1 (Crp1), which is a major isoform of mouse α-defensin, was measured by sandwich ELISA as previously described [11]. Microtiter plate wells were coated overnight at 4 °C with 100 µL of the capture antibody (77-R5) at a concentration of 1 µg/mL in 50 mM sodium carbonate-bicarbonate buffer (pH 9.6). The plate was then washed with PBS-T and blocked at 25 °C for 1 h with 200 µL of 25% Block Ace (DS Pharma Biomedical, Osaka, Japan). Next, 100 µL of Crp1 or samples were added to the wells and incubated at 25 °C for 2 h. After washing in PBS-T, 100 µL biotinylated detection antibody (77-R20, 0.5 µg/mL) was added at 25 °C for 1 h. Subsequently, the wells were incubated with 100 µL of streptavidin-horseradish peroxidase conjugate (GE Healthcare, Little Chalfont, UK) in a 1:5000 dilution at 25 °C for 1 h. After the final wash, 100 µL of TMB chromogen substrate buffer was added and incubated at 25 °C for 30 min. The reaction was stopped by adding 100 µL of 0.6 N H_2_SO_4_, and absorbance values were determined at 450 nm using a microplate reader (Multiscan FC, Thermo Fisher Scientific, Waltham, MA, USA).

### 2.6. Bactericidal Assay

The bactericidal assay was performed as previously described [1]. Secretions collected from crypts exposed to PBS, 100 µM butyric acid, and 1 µM leucine obtained in Section 2.4 were analyzed for bactericidal activity against 1 × 10^3^ colony-forming units of *Salmonella typhimurium PhoP*- (*S. typhimurium*) incubated at 37 °C for 1 h. Surviving colonies were quantified by evaluating their growth on semi-solid media at 37 °C overnight. Bacterial survival rates were determined from surviving colonies relative to the PBS control.

### 2.7. qPCR Analysis of Receptor and Transporter Gene Expression

The small intestinal crypts were resuspended in HBSS 300 U/mL collagenase (Sigma-Aldrich), 10 μM Y-27632 (Sigma-Aldrich), and 1 mM *N*-acetylcysteine (Sigma-Aldrich) at 37 °C, and shaken at 180 rpm for 5 min on a horizontal shaker (TAITEC, Kyoto, Japan). Next, 50 μg/μL DNase I (Roche, Basel, Switzerland) was added and the sample was mixed by pipetting. Cells were pelleted at 500× *g* for 5 min at 4 °C and resuspended in washing buffer (DMEM/F12, 10 μM Y-27632, 1 mM *N*-acetylcysteine). Cells were then passed through a 40-μm cell strainer (BD Falcon, Franklin Lakes, NJ, USA) and washed with washing buffer. For Paneth cell enrichment, Paneth cell granules were stained with 10 μM Zinpyr-1 (Santa Cruz Biotechnology, Dallas, TX, USA) in washing buffer at 37 °C for 10 min and washed in washing buffer. After Zinpyr-1 staining, the cells were passed through a 35-μm-pore-size filter Cell Strainer cap (BD Falcon) prior to cell sorting. Zinpyr-1^+^ cells were sorted by flow cytometry (JSAN; Bay Bioscience, Kobe, Japan). Single cells were gated by forward scatter and side scatter. Sorted cells were collected in washing buffer.

For Paneth cell isolation, Paneth cells were identified as Zinpyr-1^+^ granular cells in PBS on a poly (HEMA)-coated glass slide under a confocal microscope (A1; Nikon, Tokyo, Japan). Each Paneth cell was aspirated individually using a 50-µm glass micropipette (1-GT50S-5; NEPAGENE, Ichikawa, Japan) with micromanipulators (MN-4, MMO-202ND; NARISHIGE, Tokyo, Japan) and an electronic pipette (PicoPipet; NEPAGENE), and placed into 4 μL of lysis buffer from SingleShotTM Cell Lysis Kit (Bio-Rad Laboratories, Hercules, CA, USA) in the flat optical caps of Vari-Strip™ low profile 8 strip tubes (NIPPON Genetics, Tokyo, Japan). Each cell lysate containing 50 Paneth cells was placed in a 0.2-mL PCR tube (NIPPON Genetics).

After cell isolation, the cell lysates were incubated at 25 °C for 10 min and boiled at 75 °C for 5 min. cDNA was synthesized with the iScriptTM Advanced cDNA synthesis kit for RT-qPCR (Bio-Rad Laboratories) using 10 μL of a reaction mixture containing 4 μL of cell lysate, 2 μL of 5× iScript Advanced reaction mix, and 0.5 μL of iScript Advanced reverse transcriptase. The complete reaction was cycled at 42 °C for 30 min and at 85 °C for 5 min. Thermal cycling was performed using a Veriti Thermal cycler (Thermo Fisher Scientific). PCR primers are listed in Supplementary Table S1. The relative mRNA levels were calculated according to the 2∆Ct method, using β-actin as a reference.

### 2.8. Western Blot

Crypts were homogenized in T-PER Tissue Protein Extraction Reagent (Thermo Fisher Scientific) in the presence of a protease inhibitor cocktail (Nacalai Tesque) using a Nippi Biomasher (Nippi, Tokyo, Japan) for 1 h at 4 °C. Homogenized crypts were centrifuged at 20,000× *g* for 30 min to obtain supernatants. Protein concentrations in the supernatants were measured using a BCA protein assay kit (Thermo Fisher Scientific). Samples, including 10 mg of protein and 25 or 50 ng of mouse kidney lysate (positive control), were separated on an SDS-PAGE, following which proteins were transferred to nitrocellulose membranes. The membrane was blocked with StabilGuard (SurModics, Eden Prairie, MN, USA) for 1 h at 25 °C and then incubated at 4 °C overnight with 1 μg/mL anti-FFAR3/GPR41 (ab236654; Abcam, Cambridge, UK), anti-FFAR2/GPR43 (ABC299; Merck Millipore, Darmstadt, Germany), and anti-LAT2/Slc7a8 antibody (ab75610; Abcam) antibodies. After the membranes were washed, they were incubated for 1 h at 25 °C with goat anti-rat IgG-HRP (Imgenex, San Diego, CA, USA). After another wash, the proteins were detected using a chemiluminescent substrate (Chemi-Lumi One, Nacalai Tesque).

### 2.9. Immunofluorescence Staining

The ileum tissue from the CD1 (ICR) mice were fixed in 10% neutralized buffered formalin, embedded in paraffin, and placed on poly-L-lysine-pretreated slides. For immunofluorescent staining, after deparaffinization and rehydration, the antigens were retrieved in an autoclave at 105 °C for 20 min with Tris–EDTA (ethylenediaminetetraacetic acid) buffer (pH 9.0). After the antigen retrieval, nonspecific binding was blocked with 5% goat serum. Primary antibody reaction was performed with 1 μg/mL rat monoclonal anti-cryprdin-1 (clone: 77-R63, produced by our laboratory), 25 µg/mL anti-FFAR3/GPR41 (Abcam), 10 µg/mL anti-FFAR2/GPR43 (Merck Millipore), and 50 µg/mL anti-LAT2/Slc7a8 antibody (Abcam) diluted by PBS at 4 °C overnight. After rinsing in PBS, tissue sections were incubated with Alexa Fluor 488 goat anti-Rabbit IgG and Alexa Fluor 594 goat anti-Rat IgG (dilution 1:400, Thermo Fisher Scientific) diluted by PBS. Tissue sections were also counterstained with 5 μg/mL staining 4′,6-diamidino-2-phenylindole (DAPI, Thermo Fisher Scientific) for 5 min at 25 °C to visualize nuclei and were mounted with Aqua Poly/Mount (Polysciences, Warrington, PA, USA). Pictures were taken using a confocal microscope (A1, Nikon).

### 2.10. Quantification of Paneth Cell Granule Secretion in Enteroids

Enteroid culture was performed as previously described [18]. The pellets, as obtained in Section 2.3.2, were resuspended in HBSS supplemented with 10 μM Y-27632 (Sigma-Aldrich), the crypts were counted, and a fraction of ˃80% crypt purity was used. The fraction was centrifugated at 400× *g* for 4 min at 4 °C, and 150 crypts were resuspended in 30 μL of Matrigel (Corning, Inc., Corning, NY, USA), followed by plating in 48-well plates (Thermo Fisher Scientific). After Matrigel polymerization, 250 μL of enteroid culture medium (Advanced DMEM/F12 supplemented with penicillin/streptomycin, 10 mM HEPES, 1× GlutaMAX, 1× N2, 1× B27 (all from Thermo Fisher Scientific), and 1 μM *N*-acetylcysteine containing growth factors 50 ng/mL EGF (PeproTech, Rocky Hill, NJ, USA), 50 ng/mL HA-R-Spondin1-Fc (produced using Cultrex^®^ HA-R-Spondin1-Fc 293T Cells, Trevigen, Gaithersburg, MD, USA; #3710-001-01), and 100 ng/mL Noggin (PeproTech) were overlaid. The culture medium was changed every other day.

At day 5–7 of culture, enteroids were harvested by dissolving Matrigel with cold Advanced DMEM/F12 and transferred onto a Cell Imaging Dish (Eppendorf, Hamburg, Germany) at 150 enteroids/dish. The dish was kept on ice for 5 min to allow the enteroids to settle on the bottom of the dish. After Matrigel polymerization, enteroid culture media pre-warmed to 37 °C was added to the dish 30 min before microinjection. Binding inhibition assay (butyrate binding to Gpr41) was performed by adding culture media containing 1 mM beta-hydroxybutyrate (BHB) (Sigma-Aldrich), an antagonist to Gpr41 [19], to the dish 30 min before microinjection. For L-leucine and L-threonine injection, culture media containing Advanced DMEM/F12, which lacks individual essential amino acids (leucine or threonine, respectively) (Research Institute for the Functional Peptides, Yamagata, Japan), were added respectively, and the enteroids were microinjected with these compounds (nutrients or antagonist) after 12 h of culture. For the L-leucine transporter Slc7a8 inhibition assay, culture media (lacking L-leucine) containing 6 mM 2-amino-2-norbornane-carboxylic acid (BCH) (R & D system, Minneapolis, MN, USA), an antagonist of Slc7a8 [20], was added to the dish for 12 h before microinjection.

Microinjection was performed while scanning the enteroid at 15 frames/s by confocal microscopy in a microscope cage incubator (37 °C, 5% CO_2_, Tokken, Kashiwa, Japan). The needle (Femtotips, Eppendorf) was inserted into the enteroid by using Coarse and Fine Three-axis Oil Hydraulic Micromanipulator and One-axis Oil Hydraulic Micromanipulator (MN-4, MMO-202ND, MMO-220A, NARISHIGE), and reagents were introduced into the enteroid lumen using a Pneumatic Microinjector (IM-11-2, NARISHIGE) at a final concentration of 50 mM sodium butyrate, 1 µM L-leucine, and 1 µM L-threonine. For the Gpr41 inhibition assay, sodium butyrate and BHB were co-injected at a final concentration of 50 and 1 mM, respectively. For the Slc7a8 inhibition assay, L-leucine and BCH were co-injected at a final concentration of 1 and 6 mM, respectively.

Differential interference contrast time-lapse imaging was performed using a confocal microscope (A1, Nikon) equipped with a 0.95 NA objective lens (CFI Apo LWD 20X WI λS, Nikon) and a resonant scanner at 15 frames/s from the start of injection to 30 min after the introduction of reagents. To quantify Paneth cell granule secretion, the number of granules secreted for 30 min after the introduction of reagents was counted by observing the images displayed by the frame-by-frame playback with an image analysis software, NIS-Elements AR (Nikon). For sodium butyrate, L-leucine, and L-threonine, microinjection was performed on five enteroids, and the number of secreted granules in one experiment represents the total number of secreted granules in five microinjections. The experiment was repeated three times. For the inhibition assay, microinjection was performed on three enteroids, and the experiment was repeated four times.

### 2.11. Statistical Analysis

All statistical computations were performed using GraphPad Prism 6.07 software (GraphPad, Inc., San Diego, CA, USA). Data comparing two groups were analyzed by the two-tailed unpaired Student’s *t*-test. Data comparing several treatments were analyzed by one-way analysis of variance, followed by Tukey’s post hoc test for multiple comparisons and the Kruskal–Wallis test. Differences between groups were considered significant if *p*-values were <0.05.

## 3. Results

### 3.1. Paneth Cells Secrete α-Defensin in Response to Butyric Acid among SCFAs

**Figure 1:**
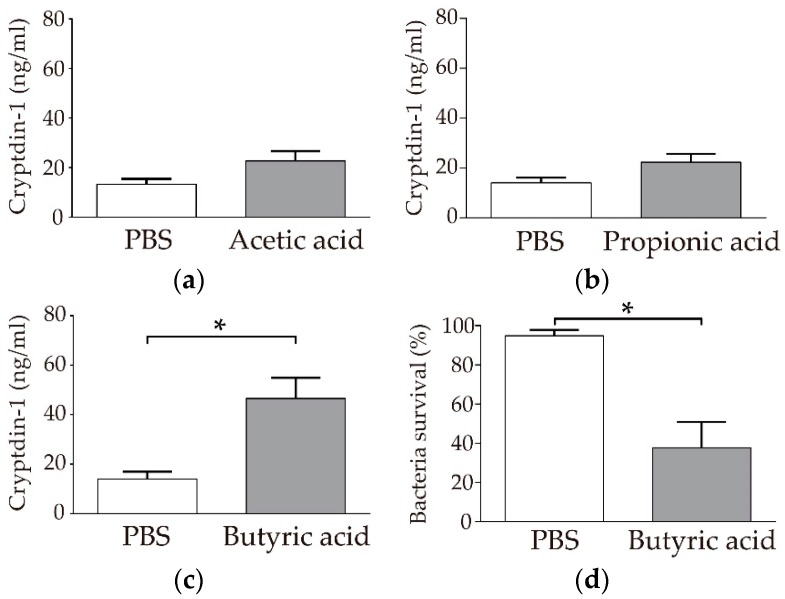
Induction of Cryptdin-1 (Crp1) secretion from Paneth cells by short-chain fatty acids using isolated crypts of the mouse small intestine: Induction of Crp1 secretion by Paneth cells in response to 100 µM (**a**) Acetic acid, (**b**) propionic acid, and (**c**) butyric acid. Data are expressed as the means ± SEM (*n* = 5). * *p* < 0.05 by Student’s *t-*test. (**d**) Bactericidal activities of Paneth cell secretions stimulated by butyric acid and PBS control against *S. typhimurium*. Data are expressed as the means ± SEM (*n* = 3). * *p* < 0.05 by Student’s *t*-test.

First, we examined whether three kinds of SCFAs, acetic acid, propionic acid, and butyric acid, induce α-defensin secretion from Paneth cells by measuring the amount of Crp1 secretion using sandwich ELISA. Butyric acid significantly induced Crp1 secretion in Paneth cells compared to in the PBS control (Figure 1c, 46.5 ± 8.4 ng/mL, *p* < 0.05). In contrast, no significant secretion was observed with either acetic acid or propionic acid compared to with PBS (Figure 1a, 22.7 ± 3.9 ng/mL, Figure 1b, 22.3 ± 3.2 ng/mL, respectively). Ascorbic acid (100 μM), a nutrient tested for comparison with SCFAs, did not induce Crp1 secretion (Supplementary Figure S1). Butyric acid further showed a tendency to induce α-defensin secretion in a concentration-dependent manner when the dose of butyric acid was changed from 1 to 100 μM (Supplementary Figure S2a). Furthermore, the collected Paneth cell secretions induced by butyric acid elicited potent bactericidal activities against *S. typhimurium* in the bactericidal assay, confirming this as the function of the secreted α-defensin (Figure 1d). Among the SCFAs tested, butyric acid induced α-defensin secretion from Paneth cells.

### 3.2. Paneth Cells Secrete α-Defensin in Response to Leucine among 20 Amino Acids

**Figure 2:**
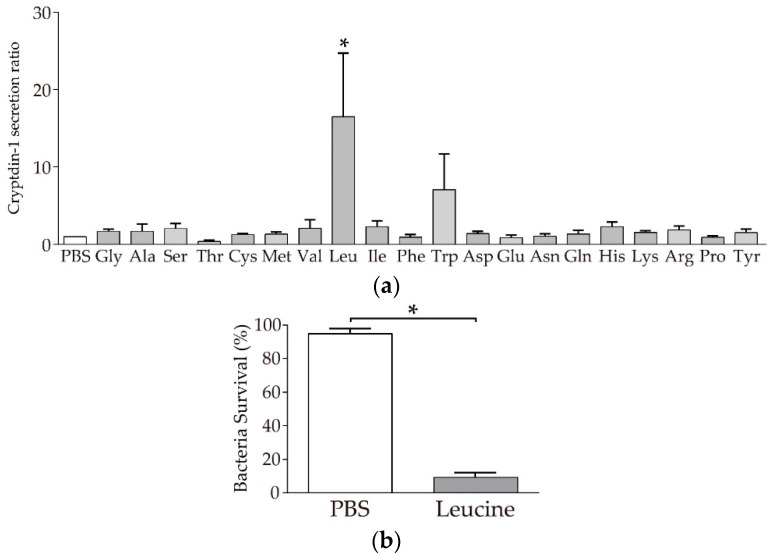
Induction of Crp1 secretion from Paneth cells by amino acids using isolated crypts of the mouse small intestine: (**a**) Induction of Crp1 secretion by Paneth cells in response to 1 µM amino acids. The results are shown as a relative ratio of Crp1 concentration compared to PBS. Data are expressed as the means ± SEM (*n* = 3–6). * *p* < 0.05 by Tukey’s multiple comparisons test. (**b**) Bactericidal activities of Paneth cell secretion stimulated by leucine and PBS control against *S. typhimurium*. Data are expressed as the means ± SEM (*n* = 3). * *p* < 0.05 by Student’s *t*-test.

Next, Crp1 secretion-inducing activities from Paneth cells were examined for all 20 amino acids. Of the 20 amino acids, leucine significantly induced Crp1 secretion compared to PBS (Figure 2a). In contrast, the remaining 19 amino acids examined did not induce Crp1 secretion. When the secretion induction activities of leucine were tested in 100 nM to 10 µM, no significant difference was observed at any of the doses despite the significant secretion induced in Paneth cells by leucine (Supplementary Figure S2b). In the bactericidal assay, the secretions collected from Paneth cells induced by leucine showed potent bactericidal activities against *S. typhimurium* (Figure 2b).

### 3.3. Paneth Cells Express Genes and Proteins for Butyric Acid Receptors and Amino Acid Transporters

**Figure 3:**
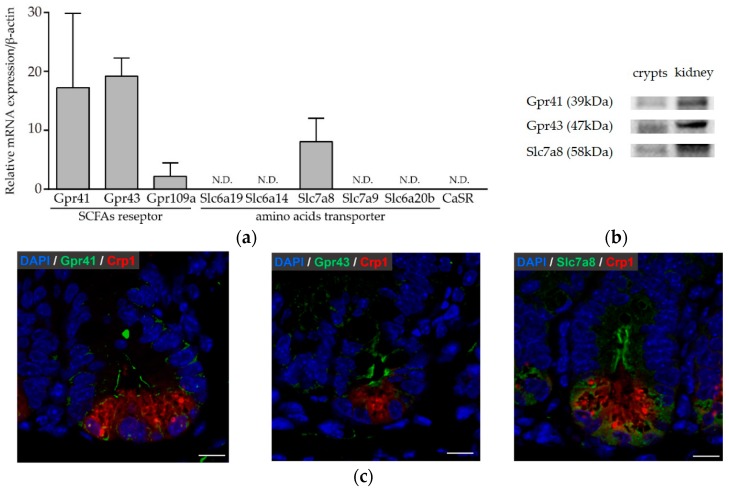
Expression of receptors and transporters recognizing short-chain fatty acids or amino acids in Paneth cells: (**a**) Relative expression of mRNA is shown by the 2∆Ct method as the means ± SEM (*n* = 3). (**b**) Western blot analysis of Gpr41, Gpr43, and Slc7a8 protein expression. Proteins were extracted from crypts of the mouse ileum and kidney. (**c**) Immunofluorescence staining of Gpr41, Gpr43, and Slc7a8 with Crp1 and 4’,6-diamidino-2-phenylindole (DAPI) in the mouse ileum. Scale bar: 10 μm.

Secretion from Paneth cells was induced by limited components, i.e., butyric acid and leucine from among the SCFAs and amino acids, respectively. As such, further analysis of whether Paneth cells express receptors and transporter that recognize butyric acid and leucine was carried out using highly purified Paneth cells. The expression of the SCFA receptor G protein-coupled receptor (GPCR), amino acid transporter solute carrier (SLC) family, and Ca^2+^-sensing receptor (CaSR) in highly purified Paneth cells was quantified by real-time PCR (Figure 3a). Paneth cells were found to express *Gpr41*, *Gpr43*, and *Gpr109a* genes, which are SCFA receptors. Paneth cells also expressed *Slc7a8* (L-type amino acid transporter-2: LAT-2) using a neutral amino acid other than proline as a substrate. These results showed that Paneth cells express *Gpr41*, *Gpr43*, and *Gpr109a*, which recognize SCFAs, and *Slc7a8*, which is an amino acid transporter, and further suggest that Paneth cells secrete α-defensin by recognizing butyric acid and leucine via these receptors and transporters. Western blots were performed for Gpr41 (FFAR3), Gpr43 (FFAR2), and Slc7a8 whose mRNA expression was observed by qRT-PCR (Figure 3a). Bands with the molecular weight of 39 kDa for Gpr41, 47 kDa for Gpr43, and 58 kDa for Slc7a8 were shown, and the expression of these proteins was confirmed (Figure 3b). Furthermore, immunohistochemical analysis revealed Paneth cells express Gpr41, Gpr43, and Slc7a8 in the mouse ileum (Figure 3c).

### 3.4. Butyric Acid and Leucine Induce Crp1 Secretion in Enteroids

**Figure 4:**
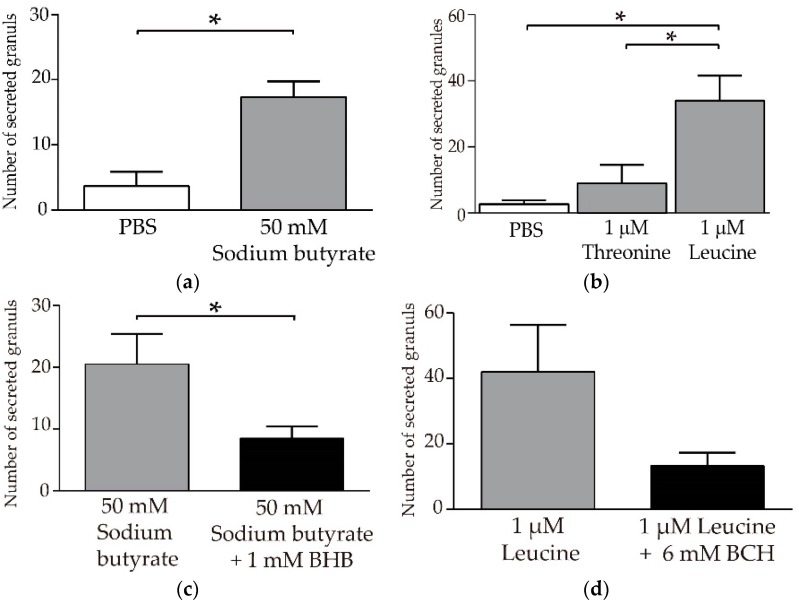
Quantification of secreted Paneth cell granules in response to butyric acid and leucine: (**a**) Enteroids at days 5 to 7 were injected with sodium butyrate (right bar, 17.3 ± 4.2) or PBS (left bar, 3.7 ± 3.8). Secreted granules were counted for 30 min after injection of reagents. Data are expressed as the total number of secreted granules from five Paneth cells ± SEM (*n* = 3). * *p* < 0.05 by Student’s *t*-test. (**b**) Secreted granules after the introduction of L-threonine (middle bar, 9.0 ± 9.6) and L-leucine (right bar, 34.0 ± 13.1) were compared to that after PBS (left bar, 2.7 ± 2.1). Data are expressed as the total number of secreted granules from five Paneth cells ± SEM (*n* = 3). * *p* < 0.05 by Tukey’s multiple comparisons test. (**c**) For the Gpr41 inhibition assay, sodium butyrate (50 mM) and beta-hydroxybutyrate (BHB) (1 mM) were co-injected. BHB significantly inhibited butyrate granules secretion. (**d**) For the Slc7a8 inhibition assay, L-leucine (1 mM leucine) and 2-amino-2-norbornane-carboxylic acid (BCH) (6 mM) were co-injected. BCH showed a tendency to inhibit granule secretion induced by leucine.

Finally, to confirm the results obtained with isolated small intestinal crypts, we further tested the stimulatory effect of butyric acid and leucine on the secretion of Paneth cell granules containing a-defensins using enteroids, which are three-dimensional cultures of small intestinal epithelial cells. Secretions were quantitatively analyzed by administering butyric acid or leucine, as well as threonine, which did not induce α-defensin secretion, compared to PBS as a control, by using the microinjection method into the lumen of enteroids (Supplementary Figure S3). We observed a significant Paneth cell granule secretion by butyric acid compared to the PBS control (Figure 4a). Leucine also significantly induced granule secretion in enteroid compared to the PBS control and threonine treatment (Figure 4b). These results reproduced and confirmed the results obtained with the isolated crypts. Furthermore, it was confirmed by using enteroids that BHB, which is an antagonist of Gpr41, significantly inhibited Paneth cell granule secretion induced by butyric acid (Figure 4c). An antagonist of Slc7a8, BCH, also inhibited leucine-induced granule secretion (Figure 4d). Moreover, butyric acid and leucine did not induce granule secretion from the basolateral side of Paneth cells in enteroid (Supplementary Methods and Supplementary Figure S4). Taken together, induction of Crp1 secretion by butyric acid and leucine resulted only through the apical stimulation of Paneth cells from the luminal side.

## 4. Discussion

This study revealed that Paneth cells recognize not only bacteria but also butyric acid and leucine and that they secrete α-defensin in response to microbial metabolites or nutrients. Furthermore, the secretions induced by butyric acid and leucine elicit bactericidal activities against pathogenic bacteria, suggesting that butyric acid and leucine as microbial metabolites or nutrients contribute to the maintenance of intestinal homeostasis by inducing α-defensin secretion from Paneth cells.

SCFAs are carboxylic acids with six or fewer carbon atoms, comprised of acetic acid, propionic acid, and butyric acid. These molecules are known to be the main metabolites produced by the intestinal microbiota from substrates, such as resistant indigestible polysaccharides derived from the diet, as well as dairy products and fermented foods [21]. SCFAs, produced by the intestinal microbiota, act as host energy sources and as signaling molecules via GPCR [22,23]. Using ligands, GPR41 (FFAR3), GPR43 (FFAR2), and GPR109a (HCA2) were identified as GPCRs for SCFAs [19,24,25]. Energy metabolism and inflammation are controlled by SCFAs via these GPCRs [26,27]. Among them, butyric acid was shown to act as an energy source for intestinal epithelial cells and activate immune functions [28,29]. It was reported that butyric acid activates Forkhead box P3 (Foxp3) transcription via histone deacetylase inhibition in the colon and induces the differentiation of naïve T cells into regulatory T cells [30]. Regarding the relationship between Paneth cells and butyric acid, it was reported that the Paneth cell number, *HD5* mRNA and *lysozyme* mRNA expression, were decreased in an amyotrophic lateral sclerosis mouse model, and were recovered by administration of 2% butyric acid in the drinking water to the same levels as those in wild-type mice following [31]. Additionally, butyric acid increases the *HD5* gene promoter activities [32]. This study indicates that butyric acid induces α-defensin secretion from Paneth cells, further suggesting the previously unknown enteric innate immune functions of butyric acid, in addition to metabolic functions. Moreover, we confirmed the granule secretion response of Paneth cells by microinjecting butyric acid into the enteroid lumen, which suggests that Paneth cells recognize butyric acid from their apical side in the small intestine to induce α-defensin secretion. Paneth cells expressed receptors, Gpr41 and Gpr43, which recognize butyric acid; this was confirmed by qPCR using highly purified Paneth cells and western blot analyses. Furthermore, an antagonist of Gpr41 significantly decreased butyric acid-induced granule secretion, indicating that Paneth cells recognize butyric acid via Gpr41 and secrete a-defensin in response. It is known that the butyric acid concentration in the human colon is around 12 o 94 mM and the butyric acid produced by intestinal bacteria is mostly metabolized by the colonic epithelial cells [33]. It has also been reported that GPCR is activated by SCFAs in the blood in the order of 10 to 100 μM in vitro [34]; thus, 100 μM to 50 mM butyric acid used in this study may have activated the GPCRs in Paneth cells to stimulate the secretion of α-defensin.

Amino acids are constituents of proteins, with 20 kinds existing in mammals. In this study, leucine out of 20 amino acids induced α-defensin secretion from Paneth cells. Leucine is the most abundant essential amino acid in human tissue, and its daily requirement is the largest [35]. Leucine is known to act as signaling molecules [36] and protein synthesis in the muscle [37], in addition to materials for protein synthesis. Leucine also decreases proteasome activity on the duodenal mucosa and activates cell proliferation [38]. Moreover, it has been reported that leucine promotes insulin secretion of pancreatic β cells and controls glucose metabolism [39,40], indicating that leucine regulates diverse functions in various cells. Leucine has been reported further to activate mechanistic target of rapamycin complex 1 (mTORC1) [41]. In addition, the Paneth cell has been known to decrease mTORC1 activity by calorie restriction and enhance stem cell expansion, suggesting leucine may be involved in mTORC1 activity of Paneth cells.

In this study, we showed that Paneth cells expressed the *Slc7a8* mRNA and protein, and BCH, an antagonist of Slc7a8 inhibited granule secretion induced by leucine, indicating that Paneth cells may recognize leucine via the amino acid transporter and secrete α-defensin. Since Slc7a8 is a transporter that widely recognizes neutral amino acids, leucine may be competing for other amino acids, such as isoleucine and valine, or recognized by other receptors or transporters. Although it is not clear why only leucine induced Paneth cell secretion among amino acids, our data suggest a new function of leucine. Further studies of the signaling pathway in Paneth cell granule secretion by leucine via transporters in the intestinal environment is needed.

Paneth cells may recognize butyric acid and leucine as feeding signals and consequently secrete α-defensin, thereby conducting surveillance in the intestine, i.e., contributing to the maintenance of intestinal homeostasis. Further and more detailed studies are needed to support this concept. In mice that have been subjected to total parenteral nutrition, Paneth cell granule numbers and reactivity to LPS are decreased compared to mice on a normal diet [42]. Furthermore, studies indicate that mice undergoing parenteral nutrition had decreased expression of *Crp4*, *Reg III*, and *lysozyme* mRNA in Paneth cells; these mice also had decreased Firmicutes, and increased Bacteroidetes, compared to mice fed on a normal diet [43]. Changes in the intestinal microbiota of these mice were similar to those in mice lacking the α-defensin-activating enzyme, matrix metalloproteinase-7. Overall, total parenteral nutrition-induced state of feeding stimuli absence in the intestine is similar to a α-defensin-deficient state; the composition of the intestinal microbiota is also similar in each state [5]. It is not likely that intestinal environmental factors, such as food and microbiota, would reach Paneth cells in the crypt bottom in a healthy state. However, food content, such as certain dietary fibers, the intestinal microbiota, and infection have been known to disrupt the mucous layer and make it thinner, so that nutrients can directly affect Paneth cells, as it is possible that butyric acid and leucine reach the cells [44,45].

**Figure 5:**
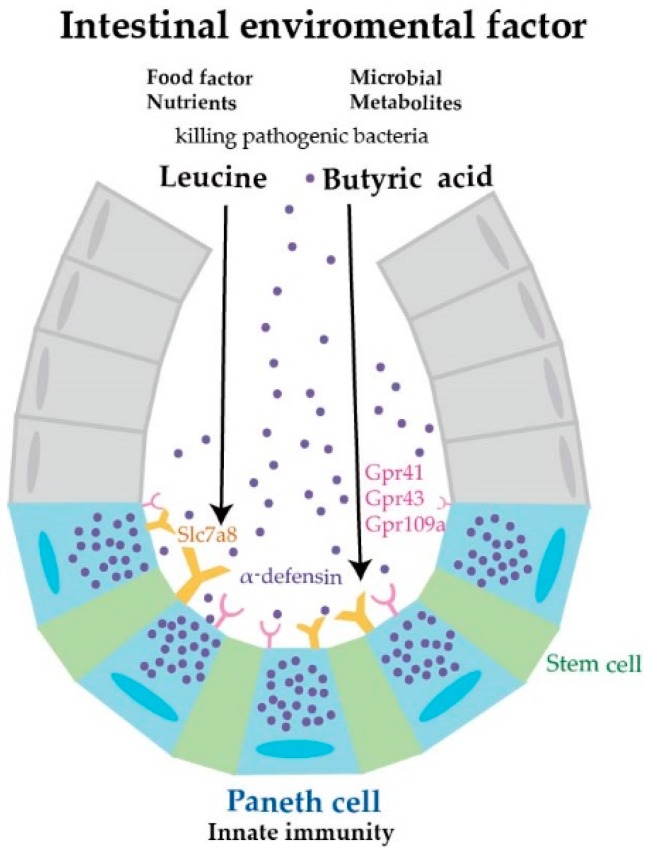
Paneth cell α-defensin secretion induced by nutrients in a possible regulation of intestinal homeostasis. This study indicates that the nutrients butyric acid and leucine induce α-defensin secretion from Paneth cells and further suggests that these nutrients perform previously unknown roles in regulating intestinal homeostasis.

Results obtained in this study are summarized in Figure 5. Butyric acid and leucine (intestinal environmental factors), nutrients from foods, and metabolites of enterobacteria induce the secretion of α-defensin from Paneth cells. Although BHB and BCH are not antagonists specific to Gpr41 or Slc7a8, our results indicate that Paneth cells may recognize and respond to butyric acid and leucine via Gpr41 and Scl7a8, respectively. This study may illustrate a new role of nutrients in the maintenance of intestinal homeostasis.

Abnormalities in α-defensin secretion are known to cause health problems and disease onset or aggravation via disruption of the intestinal environment [10]. Additionally, nucleotide-binding oligomerization domain-containing protein 2 (NOD2) mutation leads to a decrease in α-defensin production in Paneth cells in patients with Crohn’s disease [46] and the amount of immunoreactive α-defensin decreases in obese people [47]. Furthermore, in the GVHD model mice, severe dysbiosis due to Paneth cell disappearance could be reversed by administration of Wnt agonist R-Spondin1 [48,49], leading to an increase in Paneth cell numbers and recovery in α-defensin secretion, curing dysbiosis and resulting in significantly improved disease conditions [50]. Together with accumulated evidence, our data showed that certain nutritional components of foods, i.e., intestinal environmental factors, induce α-defensin secretion from Paneth cells, thus revealing a new role for nutrients, which may lead to the development of preventive methods or even therapeutics for disorders, such as lifestyle diseases. Overall, we identified a new direct innate immune function of the nutrients, butyric acid and leucine, which suggests that food contributes to the maintenance of intestinal homeostasis via α-defensin secretion by Paneth cells.

## Supplementary Materials

The following are available online at https://www.mdpi.com/2072-6643/11/11/2817/s1. Supplementary methods: Quantification of granule secretion by Paneth cell stimulated from the basolateral side. Table S1: Primer sequence list for qPCR. Figure S1: Induction of Paneth cell Crp1 secretion in response to 100 µM Ascorbic acid. Figure S2: (a) Analysis of Paneth cell Crp1 secretion in response to 1–100 µM butyric acid. (b) Analysis of Paneth cell Crp1 secretion in response to 100 nM–10 µM leucine. Figure S3: Comparison with the granule secretion before and after by SCFA or amino acid injection. Figure S4: Quantification of granule secretion by Paneth cell stimulated from the basolateral side.
