# Supplementary material for: Butyric Acid and Leucine Induce α-Defensin Secretion from Small Intestinal Paneth Cells
Source: Nutrients. 2019 Nov 18;11(11):2817. doi: 10.3390/nu11112817 (PMC6893607; doi:10.3390/nu11112817)
Supplement: Supplementary file 1 [file nutrients-11-02817-s001.zip › nutrients-584854-supplementary.pptm]

## Slide 1
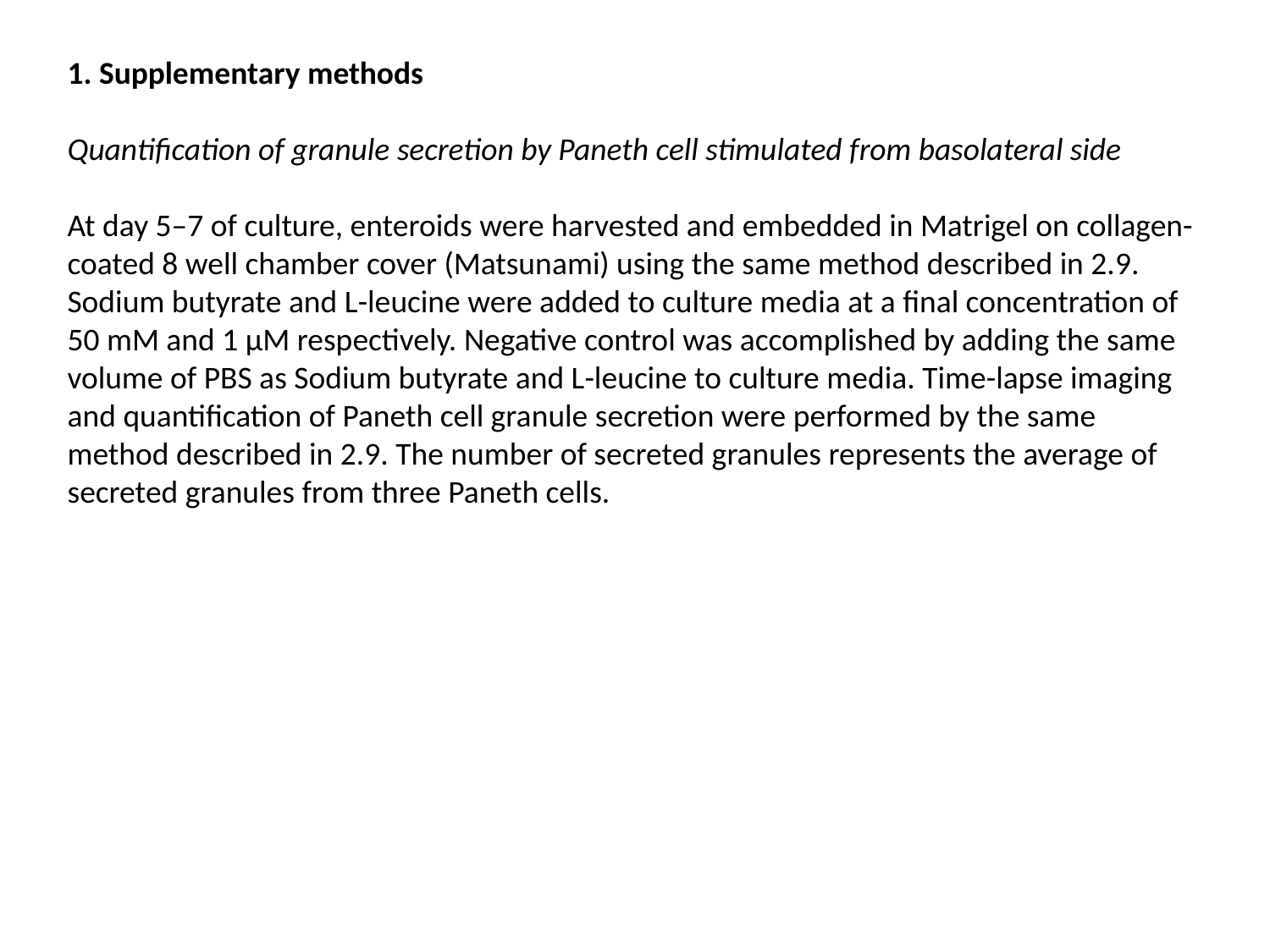

1. Supplementary methods
Quantification of granule secretion by Paneth cell stimulated from basolateral side
At day 5–7 of culture, enteroids were harvested and embedded in Matrigel on collagen-coated 8 well chamber cover (Matsunami) using the same method described in 2.9. Sodium butyrate and L-leucine were added to culture media at a final concentration of 50 mM and 1 µM respectively. Negative control was accomplished by adding the same volume of PBS as Sodium butyrate and L-leucine to culture media. Time-lapse imaging and quantification of Paneth cell granule secretion were performed by the same method described in 2.9. The number of secreted granules represents the average of secreted granules from three Paneth cells.

## Slide 2
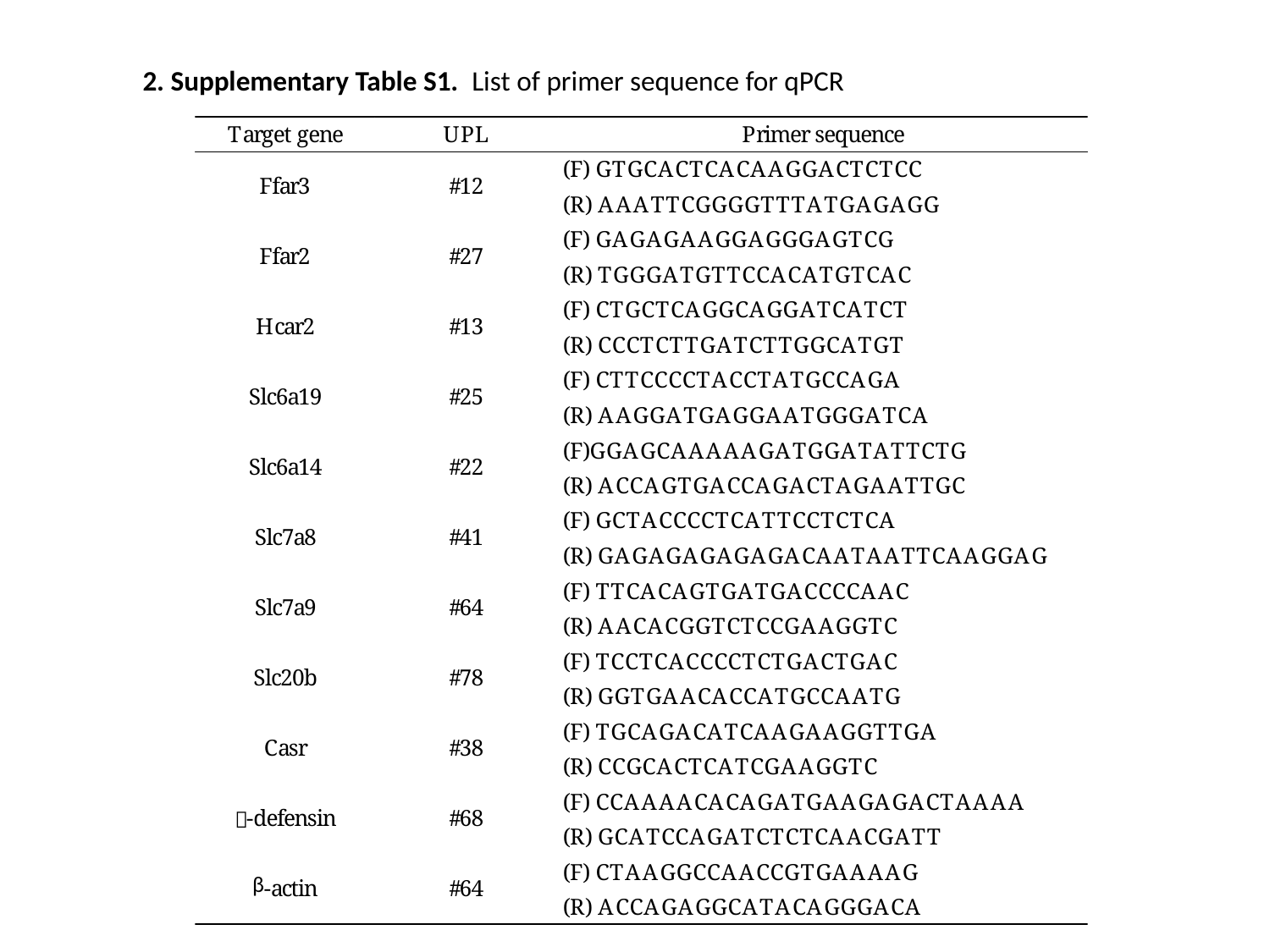

2. Supplementary Table S1. List of primer sequence for qPCR

## Slide 3
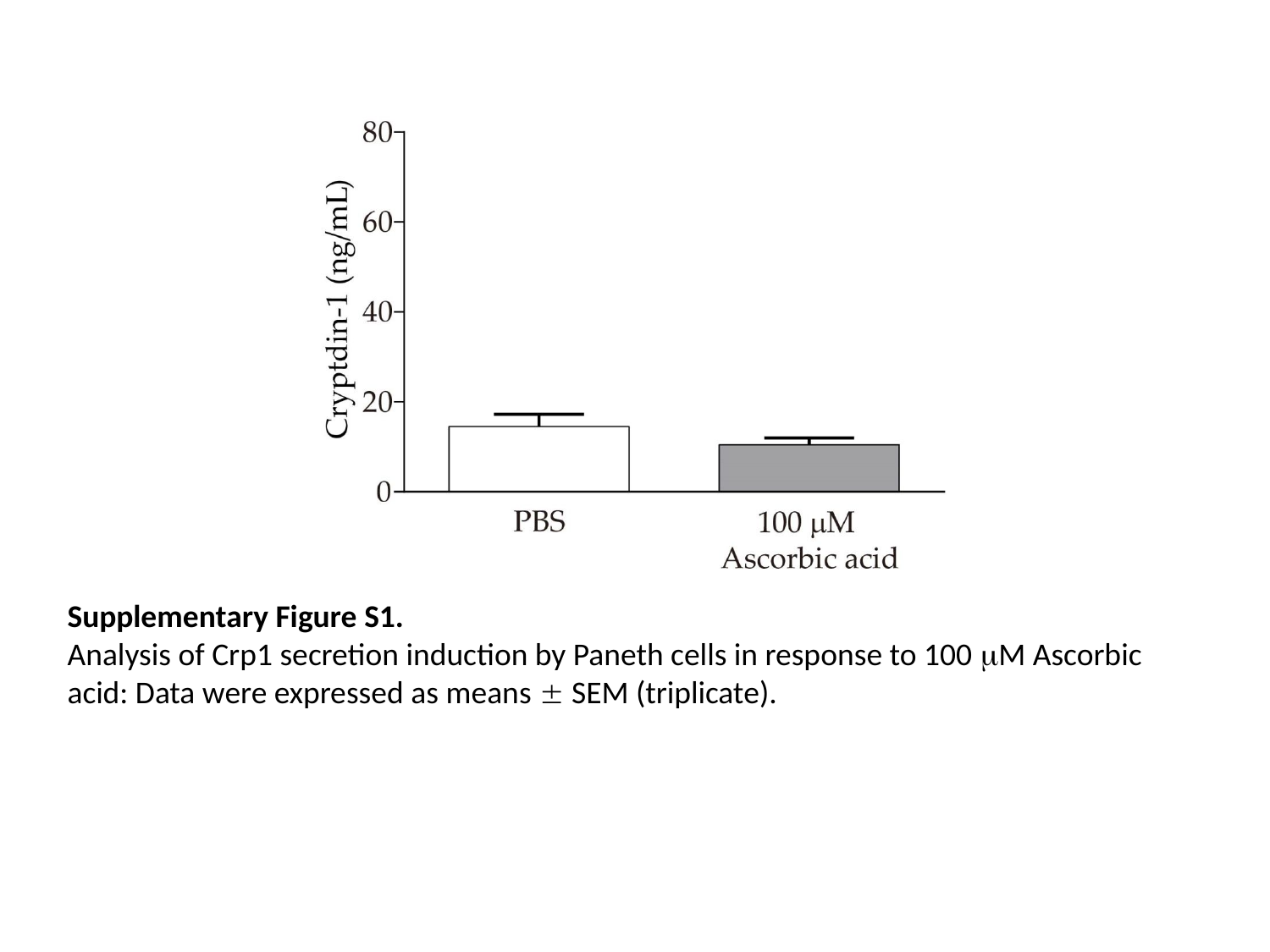

Supplementary Figure S1.
Analysis of Crp1 secretion induction by Paneth cells in response to 100 μM Ascorbic acid: Data were expressed as means ± SEM (triplicate).

## Slide 4
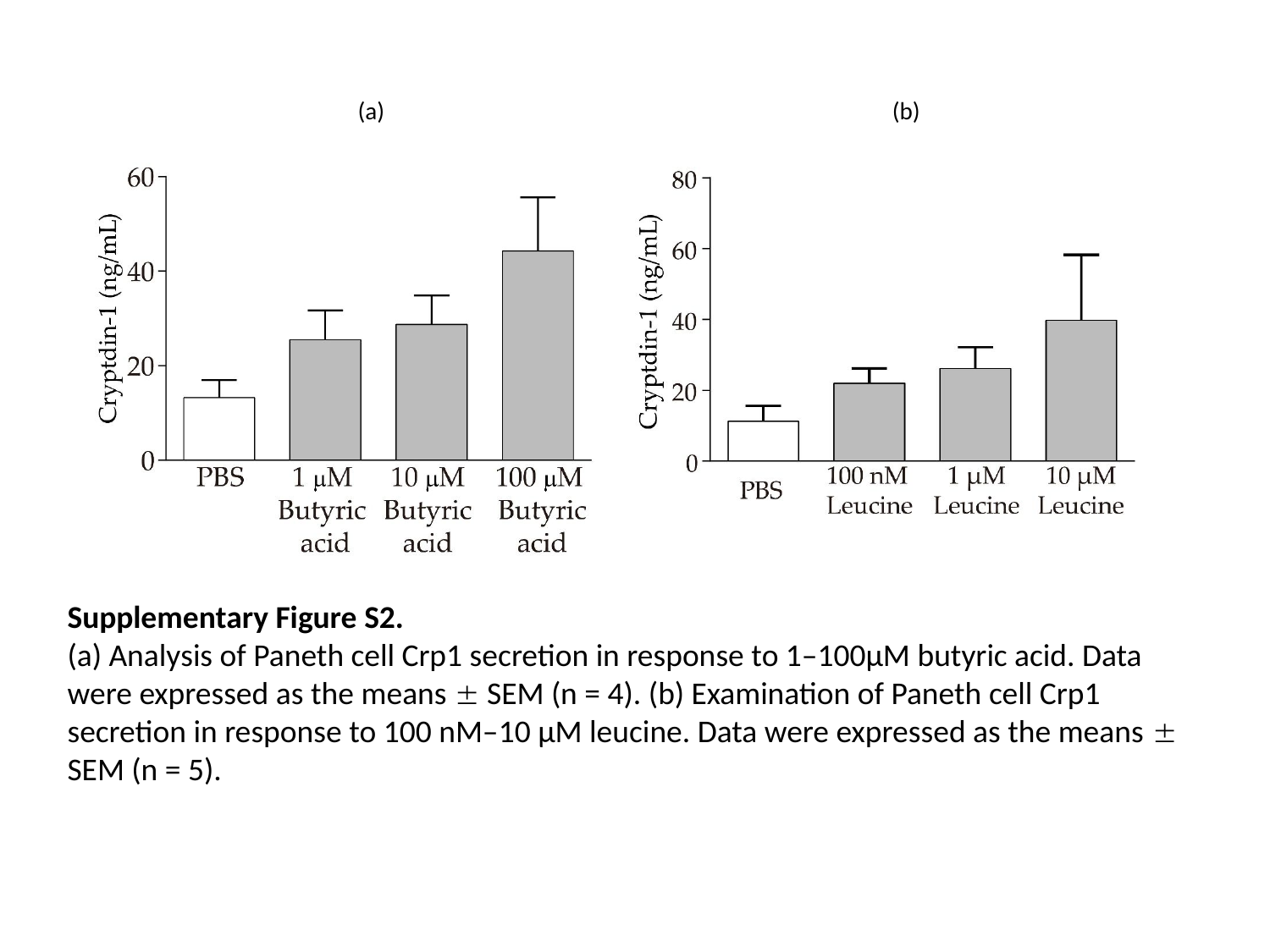

(a)
(b)
Supplementary Figure S2.
(a) Analysis of Paneth cell Crp1 secretion in response to 1–100µM butyric acid. Data were expressed as the means ± SEM (n = 4). (b) Examination of Paneth cell Crp1 secretion in response to 100 nM–10 µM leucine. Data were expressed as the means ± SEM (n = 5).

## Slide 5
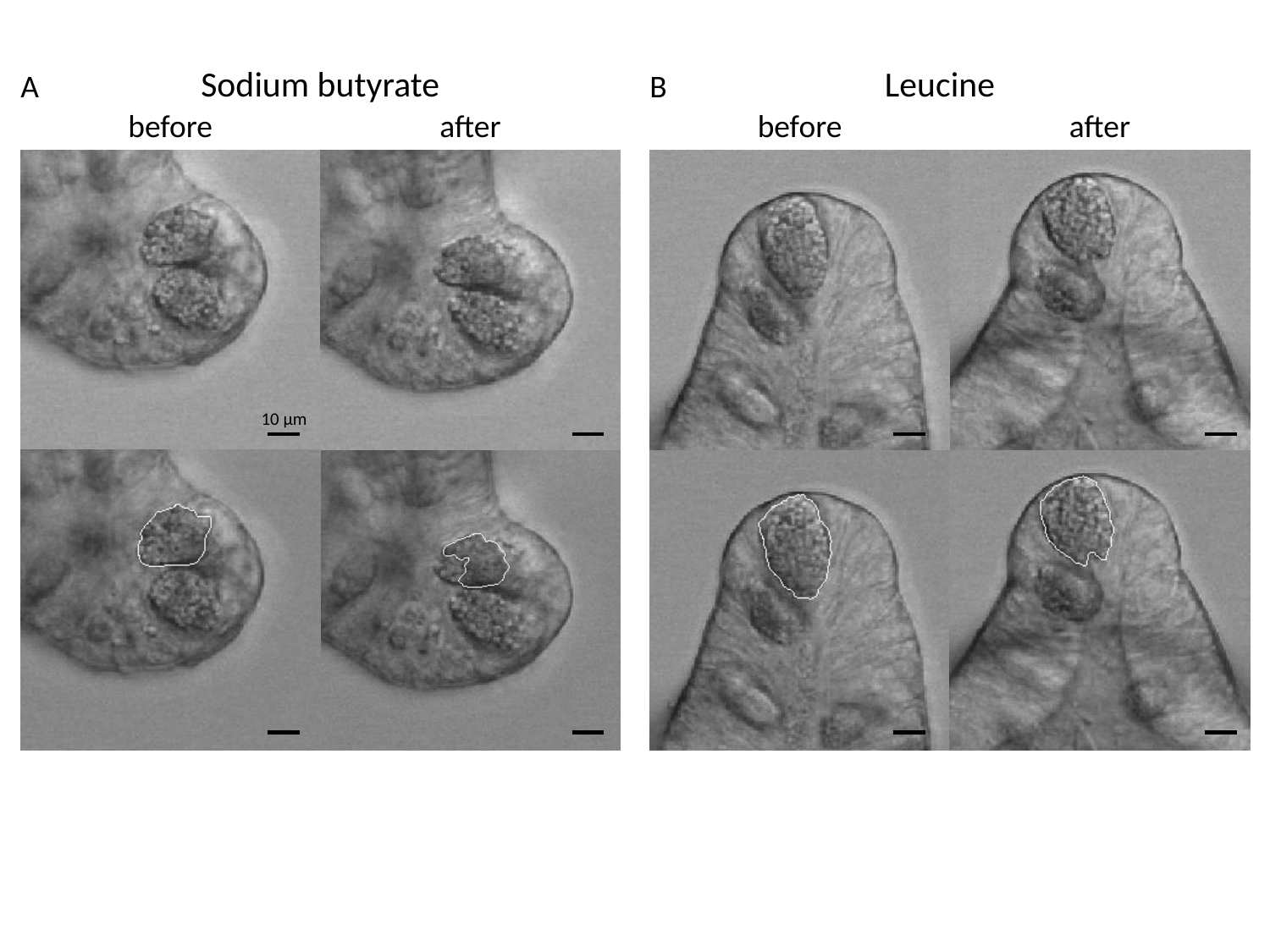

Sodium butyrate
Leucine
before
after
before
after
10 μm
A
B

## Slide 6
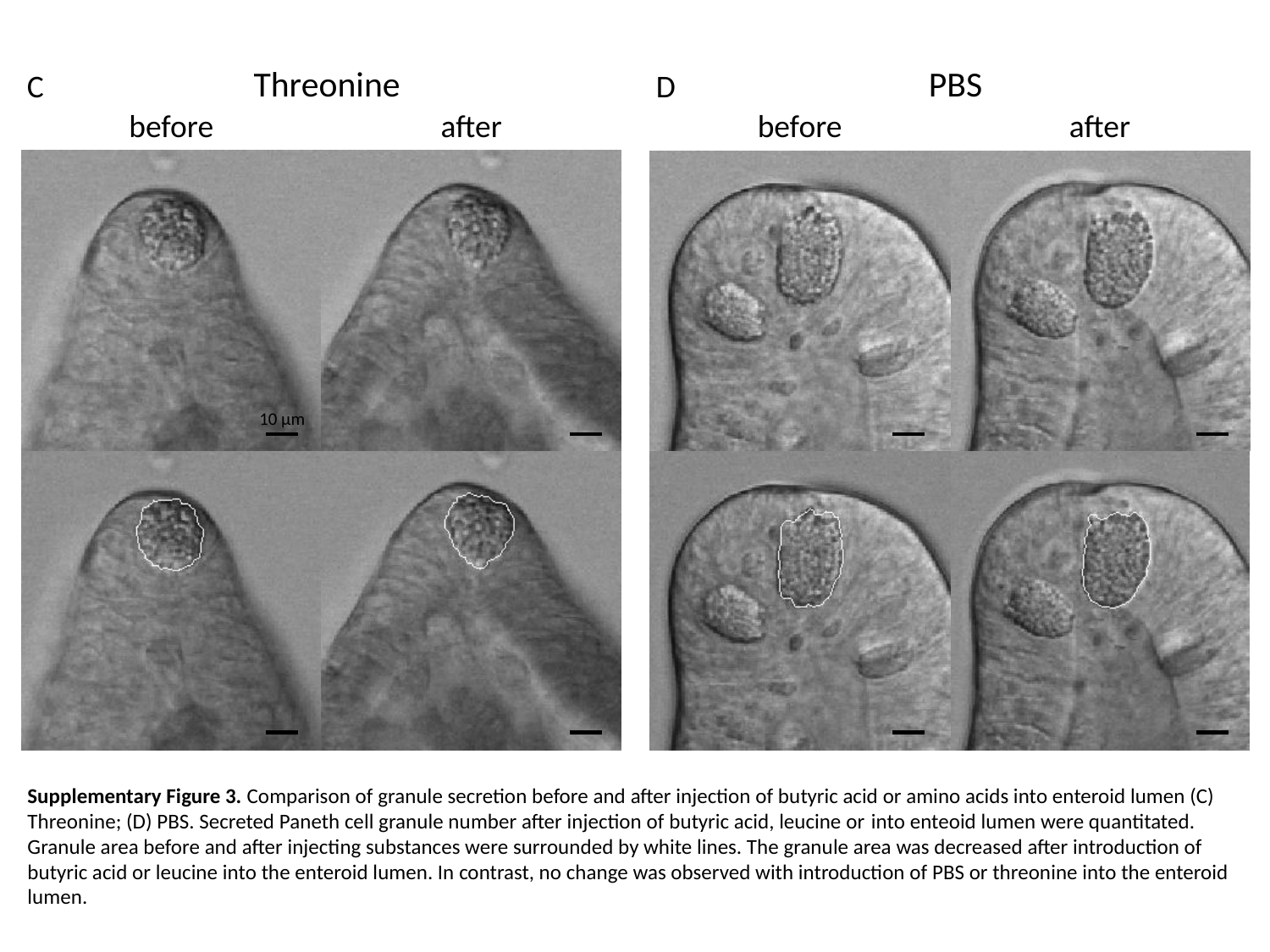

Threonine
PBS
before
after
before
after
10 μm
C
D
Supplementary Figure 3. Comparison of granule secretion before and after injection of butyric acid or amino acids into enteroid lumen (C) Threonine; (D) PBS. Secreted Paneth cell granule number after injection of butyric acid, leucine or into enteoid lumen were quantitated.
Granule area before and after injecting substances were surrounded by white lines. The granule area was decreased after introduction of butyric acid or leucine into the enteroid lumen. In contrast, no change was observed with introduction of PBS or threonine into the enteroid lumen.

## Slide 7
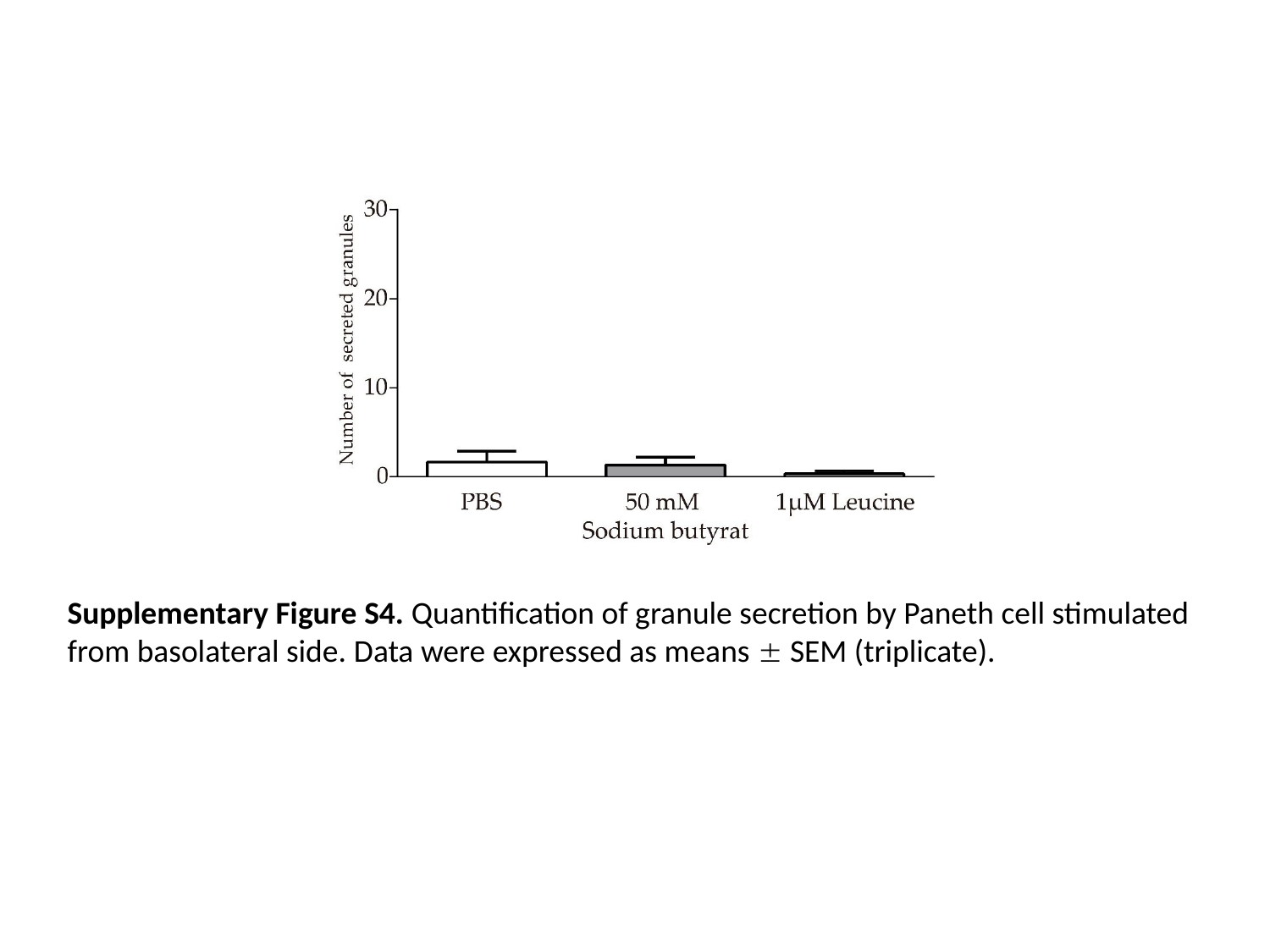

Supplementary Figure S4. Quantification of granule secretion by Paneth cell stimulated from basolateral side. Data were expressed as means ± SEM (triplicate).
